# Create Our Own Kai: A Randomised Control Trial of a Cooking Intervention with Group Interview Insights into Adolescent Cooking Behaviours

**DOI:** 10.3390/nu12030796

**Published:** 2020-03-18

**Authors:** Sarahmarie Kuroko, Katherine Black, Themis Chryssidis, Rosie Finigan, Callum Hann, Jillian Haszard, Rosalie Jackson, Katherine Mahn, Caleb Robinson, Carla Thomson, Olivia Toldi, Nicholas Scullion, Paula Skidmore

**Affiliations:** 1 Department of Human Nutrition, University of Otago, Dunedin 9016, New Zealand; sarahmarie.kuroko@gmail.com (S.K.); katherine.black@otago.ac.nz (K.B.); rosiefinigan@gmail.com (R.F.); jill.haszard@otago.ac.nz (J.H.); rosie.jackson@postgrad.otago.ac.nz (R.J.); mahn.katie@gmail.com (K.M.); caleb.robinson@postgrad.otago.ac.nz (C.R.); carla.thomson@otago.ac.nz (C.T.); oliviatoldi@gmail.com (O.T.); NRJScullion@hotmail.com (N.S.); 2 Sprout Cooking School and Health Studio, Hilton, SA 5033, Australia; themis@sprout.edu.au (T.C.); callum@sprout.edu.au (C.H.); 3 Department of Medicine, University of Otago, Christchurch 8140, New Zealand

**Keywords:** adolescent, cooking intervention, RCT, mental well-being, cooking self-efficacy

## Abstract

Cooking is frequently associated with a healthier diet, however few youth cooking intervention studies have used control groups or follow-ups. Additionally, although cooking is associated with better mental well-being among adolescents, this has not been examined experimentally. This randomised controlled trial investigated whether a five-day intensive holiday cooking program, followed by six weeks of weekly meal kits with Facebook support groups, affected the cooking-related outcomes, diet quality and mental well-being among adolescents, with a 12-month follow-up. Adolescents aged 12–15 years (intervention: *n* = 91, 60% female; control: *n* = 27, 78% female) completed baseline, post-intervention and 12-month follow-up anthropometric measures, and questionnaire measures of mental well-being, diet quality and cooking attitudes, self-efficacy and behaviours. The intervention group’s post-intervention outcomes improved significantly more for mental well-being, diet quality, helping make dinner, cooking self-efficacy and positive cooking attitude, however body mass index (BMI) z-scores also increased. Differences were maintained at 12 months for self-efficacy only. Group interviews showed that participants’ cooking behaviours were strongly influenced by family factors. Adolescent cooking interventions may have many short-term benefits, however cooking self-efficacy appears most responsive and stable over time. Effects on BMI need further investigation. Family factors influence whether and what adolescents cook post-intervention.

## 1. Introduction

Cooking skills are a resource for health [1]. Home-made meals tend to be healthier than ready-made or store-bought alternatives, and cooking interventions can give people practical tools to integrate healthier eating messages into their daily lives [1,2,3,4]. Helping with meal preparation has been associated with better diet quality among children and adolescents [5]. Growing attention is being directed at improving the diets of adolescents through cooking education, as a characteristic of adolescence is growing independence over all areas of their lives, including eating habits [6]. Diet quality typically declines during this time, yet ensuring a solid foundation in cooking as food autonomy grows could facilitate healthier eating during adolescence and beyond [1]. According to social cognitive theory (SCT), people are more likely to engage in healthy behaviours such as cooking if they have good self-efficacy for the behaviour, which is most effectively developed through practical experience [7]. However, there is concern that adolescents in the 21st century have less opportunities at home and school to learn to cook than previous generations [8]. Additionally, a proposed “culinary transition” in Western countries towards greater ready-made input into meals may mean adolescents are less exposed to cooking from basic ingredients [9]. Hands-on cooking in conjunction with nutrition education, also known as culinary nutrition, is a potential solution to ensuring adolescents have the knowledge and self-efficacy to prepare healthy meals [1]. Growing responsibility may also mean they are more likely to have opportunities at home to put their cooking skills into action.

Although rhetoric around the importance of formal cooking education (for girls) has been around since the early 20th century, scientifically evaluating cooking interventions is an emerging research area [10,11]. Many existing studies with adolescents (11–19 years [12]) have had small sample sizes with limited statistical power to detect changes, often being at feasibility or pilot stages [13,14,15,16,17,18,19,20,21]. Most studies in this age group have had prospective cohort designs [13,14,15,16,17,18,19,20,21,22,23,24,25,26], with experimental designs tending to be quasi-experimental [27,28,29,30] rather than randomised [31,32]. Few studies have looked beyond immediately post-intervention to assess longer-term effectiveness, with those that did having follow-ups of no more than six months [19,25,27,28,31]. Only one study has included an experimental design in conjunction with a follow-up [27]. Furthermore, programme characteristics have varied widely (such as the amount of time spent cooking) along with evaluation methods (such as the level of detail of dietary questionnaires) [11].

Cooking intervention research is looking beyond diet- and cooking-related outcomes to psychosocial implications [11,26,33]. Adolescent cooking ability has observationally been associated with greater mental well-being and family connectedness, along with fewer depressive symptoms [34], however this is yet to be investigated in an experimental setting. This deserves further investigation, as mental well-being typically declines during adolescence, and improving adolescent mental health is a global priority [12,35].

Adolescent cooking interventions have typically been based at school, or as an after-school or holiday activity rather than in the home [11], yet home dynamics surrounding adolescents’ food behaviours are complex, and parents remain “gatekeepers” to the kitchen [13,36,37,38]. Some programmes have encouraged family buy-in or involvement through inviting parents to a lunch cooked by their adolescents on the final day [14,15,16,17,20], having participants take cooked dishes home to share [38], providing ingredients to remake dishes at home [24], or providing meal kits (without cooking lessons) for families to prepare together [26,39]. The potential for interventions to build in support strategies which help adolescents apply their new skills at home could be further utilized, such as by combining some of these strategies and having a dedicated support phase after the teaching kitchen component. Furthermore, adolescent cooking dynamics could be better understood, as facilitators and barriers to adolescents cooking at home have been qualitatively touched on in the context of collecting cooking intervention feedback [15,19,21,38,39,40], or understanding food choices and behaviours more broadly [41,42,43,44,45], rather than focusing on adolescent cooking behaviours [46].

The aim of the COOK (Create Our Own Kai) Study was to determine the effects of a cooking intervention on mental well-being, diet quality and cooking-related outcomes, with a 12-month follow-up. We hypothesised that all outcomes would improve in the intervention group relative to the control group at post-intervention and that a difference would remain at follow-up. Further aims of this study were to assess the feasibility of a support phase which followed the cooking classes component, in order to facilitate participants in building cooking habits at home, as well as to explore factors which influenced adolescents’ home cooking involvement through group interviews.

## 2. Materials and Methods

### 2.1. Study Design

**Figure 1:**
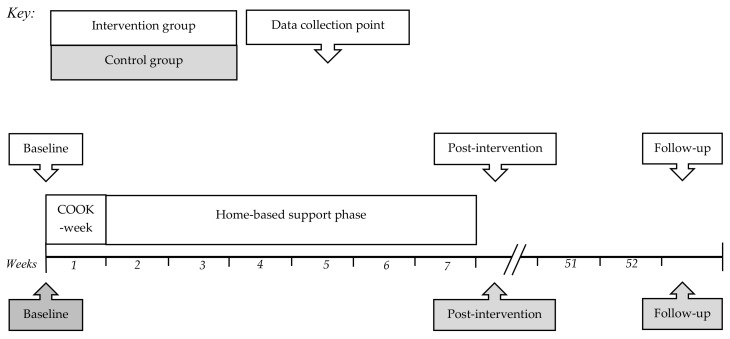
Timeline for streams’ A, B, C and D of the COOK Study.

The COOK Study was a parallel RCT of a cooking intervention (Figure 1). To keep classes at a manageable size, participants were broken up into one of four streams (Streams A, B, C and D), then within each stream allocated to the intervention or control condition. The intervention arm had two phases. Phase one (COOK week) was an intensive five-day practical cooking program during school holidays. Phase two (support phase) was a home-based, social media-led six-week period, when participants received weekly meal kits. Control participants completed study measurements only. Questionnaire and anthropometric measures were taken post-allocation at baseline, post-intervention (seven weeks from baseline) and follow-up (12 months from baseline). Participants received a $20 voucher (Foodstuffs, New World) for each set of measures they completed, except for intervention participants’ baseline measures, which were part of COOK week. Voluntary group interviews were held at post-intervention and follow-up after quantitative data collection had finished. This trial was prospectively registered (ACTRN12616001664437) and ethical approval was provided for all parts of the study by the Otago University Human Ethics Committee (16/126).

### 2.2. Recruitment and Allocation

**Figure 2:**
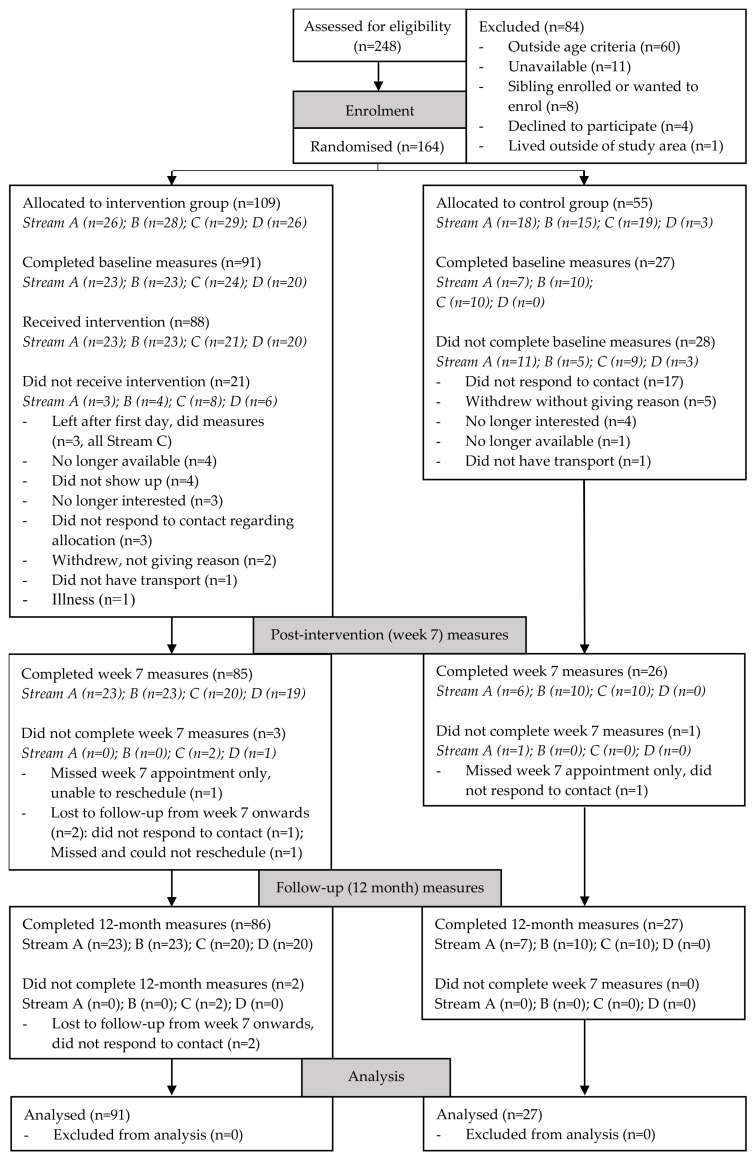
Participant flow during the COOK Study.

Adolescents in their first two years of high school (mostly 12–15 years old), residing in Dunedin, New Zealand, were recruited via social media, posters and word of mouth. Exclusion criteria were having another sibling enrolled in the study, or a disability that prevented them from working safely in a kitchen. Assent and written conformed consent were collected from participants and their legal guardians, respectively. We aimed to recruit 200 participants. Stratified randomisation per stream was used for streams A, B and C, which began during January 2017, while simple randomisation was used for stream D, which started in July 2017. The intervention was designed for a class size of between 24 to 26 participants, which we held constant across streams so that participants in different streams had similar experiences. To achieve this, despite participant numbers varying per stream, we preferentially randomised to the intervention group. Within each stream, enough participants per stream to fill the intervention classes were randomly assigned to the intervention group (with two or three extra as back-up in case of drop-outs), and any remaining were assigned to the control group. We therefore aimed to recruit 100 participants for each condition, so that the allocation ratio would be approximately 1:1 for the intervention and control conditions while maintaining the class size. Randomisation was carried out separately for each stream by two researchers. Papers folded so that the writing (“intervention” or “control”) was hidden were drawn out of a container, while working sequentially down the participant list for that stream. Each stream’s container held between 26 and 29 papers reading “intervention” and enough “control” papers for the total number of papers in the container to match the total number of participants in that stream (Figure 2).

### 2.3. Intervention Part 1: COOK Week

COOK week was adapted from a commercial cooking programme developed for Australian school children by Sprout (Sprout Cooking School, Adelaide, Australia). This program was modified in order to make sure it was suited to the New Zealand context, and appropriate according to Māori cultural practices (such as not allowing sitting on tables) [47]. COOK week was piloted in December 2016 [15], and some logistical aspects were modified for the main study based on participant feedback on the pilot study, such as giving participants trays of ingredients to save time lining up for items.

COOK week ran Monday to Friday from 9 a.m. until approximately 3:15 p.m., in local educational facilities’ teaching kitchens. Participants worked in pairs for the week, with two pairs per workstation. Where possible, those with similar dietary requirements or food dislikes were paired. An allergen management plan was implemented. All sessions were co-delivered by a New Zealand-registered dietitian and qualified chef, supported by research assistants. Presentations and discussions between cooking sessions included kitchen and food safety, clear communication, nutrition, food waste, seasonal and local produce, budgeting, writing recipes and shopping lists, and budgeting, and selecting foods at the supermarket. Participants cooked three dishes most days. Recipes, which costed approximately NZ$5 per person per meal, encouraged participants to experience a wide range of different foods, inspired from many world cuisines and using a variety of ingredients. They contained no added salt, apart from a couple of dishes with soy sauce; flavour was developed through herbs, spices, citrus and vinegar. Vegetables were plentiful in savoury dishes, and desserts were mostly fruit-based with small amounts of sugar when appropriate. Instructors gave a full demonstration first for all dishes except Thursday’s, which was prepared without demonstrations so that participants could practice following a recipe themselves. Each participant invited a family member for a two-course lunch on the final Friday that was budgeted for, designed, and prepared by participants.

### 2.4. Intervention Part 2: Support Phase 

Participants received six weekly bags with a recipe and ingredients to make a family meal (meal kits). Meal kits costed NZ$12 or less, were designed to comfortably feed a family of four, and only required basic kitchen equipment. Collectively, these tasks reinforced a range of skills used during COOK week, and some new techniques were also introduced (e.g., making white sauce). Support from research assistants and contact with classmates were provided via private Facebook groups. Participants were encouraged to post photos of any cooking they did for the rest of the study. During the support phase, there were weekly prizes for whoever put up the most cooking posts, and spot prizes inside the meal kits. 

Throughout the study the control group were not asked to do anything except fill out the same questionnaires as the intervention group, at the same time points. 

### 2.5. Data Collection

Baseline measures were collected either in the teaching facilities (intervention group) or during appointments at a research clinic (control group). Postintervention measures for both groups were completed during clinic appointments for the three summer streams, but this was modified for the July stream (stream D) due to logistical difficulties experienced with the earlier streams. Stream D was given their questionnaires with their final meal kit, to complete at home and bring back for their anthropometry appointment—appointments could then be considerably shorter, which parents found easier. This was repeated for all 12-month follow-up collections by mailing out questionnaires in advance.

### 2.6. Questionnaires

Participants indicated with questionnaires, their date of birth, age and sex. Ethnicity was collected and coded using the NZ census method [48]. Socio-economic status (SES) was estimated from participants’ residential addresses using the NZDep2013 system of categorising neighbourhood deprivation level [49], then categorised as “low” (NZDep2013 scores 8–10), “middle” (4–7) and “high” (1–3).

Mental well-being was measured with the five-item World Health Organisation-5 (WHO-5) Well-being Index, with items summed and converted to a percentage of the maximum possible score [50]. Dietary intakes were assessed using the 72-item non-quantitative NZ Adolescents’ Food Frequency Questionnaire (NZAFFQ). A diet quality index based on the adequacy and variety of foods within major food groups according the NZAFFQ’s scores has been developed, giving a more integrated way of interpreting food frequency data than evaluating by food groups alone. Both the FFQ and its diet quality index (NZDQI-A) have been tested for reliability and validity [51,52]. 

Cooking frequency was investigated, asking: “how many times a week do you help prepare food for your evening meal?”, adapted from Project EAT [53], and “in a normal week, how often do you prepare and cook a main meal from basic ingredients, for example, spaghetti bolognaise, starting with raw mince and tomatoes?”, adapted from the CookWell program [54]. Both items were scored out of seven, for the number of days per week respondents usually cooked. 

Relevant cooking self-efficacy items were selected from questionnaires validated with adults, which we also considered suitable for adolescents [54,55]. General cooking self-efficacy (general self-efficacy) was evaluated using four items from the CookWell programme [54]. Participants indicated on a 7-point Likert scale how confident they felt about “being able to cook from basic ingredients”, “following a simple recipe”, “tasting foods you’ve not eaten before”, and “preparing and cooking new foods and recipes”. Self-efficacy for specific cooking preparation tasks and techniques (specific self-efficacy) items were adapted from Cooking with a Chef [55], with two additional items regarding preparing meat and sauces adapted from the UK’s National Diet and Nutrition Survey to the same format [56]. On a 5-point Likert scale, participants indicated how confident they felt about performing 18 skills—12 discrete technical skills (e.g., simmering) and six broader preparation skills (like preparing fresh and frozen vegetables). General and specific self-efficacy scores were created by summing items and converting to a percentage of the maximum possible score. 

The cooking attitude scale was created especially for the COOK Study and tested for clarity with a convenience sample of adolescents prior to the COOK pilot [15]. Participants responded to five positively worded statements around cooking, such as “I enjoy preparing food even if it takes a lot of time” on a 5-point Likert scale. Responses were then converted to a percentage of the maximum possible score.

### 2.7. Anthropometry

Anthropometric measurements were taken by trained research assistants in private. Participants wore light clothing and had bare feet. Height was recorded to the nearest 0.1 cm while participants stood straight with their heads in the Frankfort plane, using a portable stadiometer (Wedderburn portable height rod: WS-HRP, Dunedin). Measurements were repeated, a third was taken if the first two differed by 0.5 cm or more, and height was calculated as the mean of the closest two measurements. Mass was measured to the nearest 0.1 kg using bio-electrical impedance analysis scales (C418, Tanita, Tokyo, Japan). World Health Organisation age- and sex-specific z-scores were applied to body mass index (BMI; kg/m^2^) scores, which were then categorised as underweight (BMI z-score < −2), normal weight (BMI z-score < 1), overweight (BMI z-score ≥ 1 and <2), and obese (BMI z-score ≥ 2) [57]. Due to small numbers of underweight and obese participants, categories were collapsed into “normal/underweight” and “overweight/obese”.

### 2.8. Data Entry and Statistics

Quantitative data were independently entered twice into Excel (Microsoft Office 10, Washington, DC, USA). Stata (version 15.0, StataCorp, College Station, TX, USA) was used for identifying discrepancies between entries, missing values, outliers and random values, along with running all statistical tests. Worst-case scenarios were used when participants had selected more than one option. Missing values were imputed for mental well-being, cooking self-efficacy and cooking attitude scales, using the mean of non-missing scale items.

Analyses followed intention-to-treat principles. Between-group differences were estimated using mixed regression models, with participant and intervention stream as nested random effects. An interaction term between time and randomised group was included. Mean differences, 95% confidence intervals (95% CIs), and *p*-values were calculated. Means and standard deviations for within-group changes in outcomes between time-points were calculated for descriptive purposes. 

### 2.9. Group Interviews

Group interview participation was optional. Attendees were given afternoon tea and $10 supermarket vouchers for participating. To protect the integrity of questionnaire data, interviews only took place once quantitative data collection at each point had completely finished, therefore interviews were held some time after the main data collection time-points of 7 weeks and 12 months. One interview was undertaken with stream D intervention participants 10 weeks from baseline, for feedback on the intervention’s support phase and to inform interview questions at follow-up. End-of-study interviews were held with streams A–C approximately 18 months from baseline, separately for control and intervention participants, investigating experiences, barriers and facilitators for cooking at home. 

The facilitator worked through question schedules, while an assistant took notes. End-of-study question schedules were the same for control and intervention groups, except for additional questions asking intervention participants to compare current situations with the end of the COOK programme. Sessions were audio-recorded, transcribed ad verbatim, then responses were listed and summarised under each topic. Formal focus groups and thematic analyses were not undertaken, as the primary objectives of this study were quantitative. Responses from control and intervention participants were summarised together (except for intervention-specific questions), as answers were similar.

## 3. Results

### 3.1. Participant Summary

Participant flow is described in Figure 2. A total of 164 participants enrolled in the study (44, 43, 48 and 29 enrolling in streams A to D, respectively), of which 109 were assigned to the intervention in order to maintain a consistent intervention class size across streams. There were 46 withdrawals after allocation, with a significantly greater drop-out rate among the control group (51% vs. 17%, χ^2^ = 19.2, *p* < 0.001). All participants completing any baseline measures were included in the regression analyses, giving a total of 27 control and 91 intervention participants. The mean actual follow-up length was 55.0 ± 2.7 weeks. Demographic and baseline characteristics are described in Table 1. Participants had a mean age of 13.6 ± 0.8 years and 64.4% were female.

### 3.2. Postintervention and Follow-up Results

The intervention group’s mental well-being scores increased by an average of three points more from baseline at post-intervention (*p* = 0.005), and diet quality index scores by four points (*p* = 0.041; both scale ranges: 0–100), but neither changes were significantly different from the control group at 12 months (Table 2). At post-intervention, BMI z-scores increased by 0.08 more from baseline among the intervention group (*p* = 0.006), with no difference between groups at follow-up (Table 2).

At post-intervention, the frequency of helping prepare an evening meal increased by a mean 0.4 evenings per week (*p* = 0.001) among the intervention group, however there was no significant difference for cooking a main meal from basic ingredients, and neither of the cooking frequency outcomes differed between groups at follow-up (Table 3). Both cooking self-efficacy measures increased significantly more for the intervention group at post-intervention, remaining higher than the control group at follow-up (all *p* < 0.001). Effect sizes were larger for self-efficacy for specific tasks and techniques than for general cooking self-efficacy. The intervention group’s attitude scores increased by a mean 11 points more at 7 weeks (*p* < 0.001), with no difference at follow-up. 

### 3.3. Group Interviews

Thirty-three participants took part in group interviews (12 male). The single post-intervention interview was with intervention participants only (*n* = 15; 6 male). End-of-study interviews consisted of two with intervention (*n* = 13; 4 male) and one with control participants (*n* = 5, 2 male). End-of-study interviewees were aged 14 to 17 years.

#### 3.3.1. Intervention Experience and Perceived Changes 

During the post-intervention interview, participants were asked what they had expected of COOK week and how this compared with their experience. Expectations were exceeded, with most agreeing with two who said “I thought it was gonna be boring lectures and me just being awkward around other people, but it was, like, a lot more fun” and that “it just rocketed away, sky-rocketed”. Participants had not expected to do as much practical cooking and particularly enjoyed this, as well as getting to choose what to make on the final day. It was common to feel “a little bit worried and nervous” or “whakamā” (embarrassed) when finding out they would be paired with someone they did not know. However, this was usually a positive experience and many participants indicated that meeting new people and making friends was an aspect of COOK week they highly valued (“you can, like, um, learn new things, um, finding friends”). 

Participants described enjoying cooking more and feeling more confident in the kitchen during the post-intervention interview, especially as they had a better understanding of “how things fit together”. Most reported cooking about once a week. They thought they were cooking more often than before the programme, particularly for main meals (“[before] I’d be a bit scared of, um, cooking my meals because I didn’t know how they would turn out. But now, since I’m okay with doing it, it’s good”). Cooking more was partly attributed to the meal kits, which two participants mentioned as helpful for providing free food, although the main benefit for most was “not having to, like, choose the meal you’re making as well”. Other reasons for cooking more often were because they were enjoying it more, felt more confident, and parents knew that they were capable.

While making the meal kits, participants felt generally confident to substitute ingredients (such as for accommodating family preferences) and to bulk recipes out as needed (“yeah, you just chuck it in”). Recipes were more useful if they were easy to modify; harder or less enjoyable recipes had multiple components or longer cooking times. The main stressors were around getting familiar with new recipes and being in the kitchen, along with family factors. Widespread concerns were burning things, along with cutting onions, which spurred innovations (such as using goggles) and parental assistance. Parent involvement could also be a hinderance, with one participant saying parents telling them to do things differently to the recipe was confusing (“my mum was constantly, constantly trying to, sorta, not exactly change the recipe, but she’d just, like, say ‘oh no no no, don’t try that, try doing this’,…, when it worked out in a bad way, she’d say ‘well why’d you try that? You should have gone with this’”). Pressure from family members to hurry because they were hungry was also stressful.

Most parents were happy to have someone else cook (“mum liked having free food, and also having me cook it for her”). Family members did not like some dishes, particularly siblings, who would refuse to eat it, although there were also reports of siblings unexpectedly enjoying something. Participants felt more optimistic about the next meal kit if their family enjoyed the last one or would eat anything, but overall, participants enjoyed cooking and parents appreciated their teens’ effort and capability (“mum has asked me to remake some of them while she’s on holiday”). A few participants said their siblings were cooking more, either helping or getting competitive (“my older brother started to cook, just to make me look bad”), or parents now wanted siblings to also cook once a week. 

During end-of-study interviews, intervention participants generally felt their interest in cooking was “about the same” as just after the support phase, but actual cooking behaviours were seen to have changed. Some felt they cooked more, or more often for their families, and that their skills continued improving. Two agreed that “straight after the course I probably did a bit more than I do now, well at least different dishes but as time went on I probably went back to the dishes I was more used to cooking”. One described the extra cooking from the meal kits being prolonged by a commercial meal kit service, which declined once the subscription was cancelled. Participants did not seem to lack perceived cooking ability—when asked if there was anything they wanted to make but had not, most answers were for gourmet and specialty dishes, such as sous vide or baked Alaska. 

#### 3.3.2. Adolescent Cooking Practices 

Control and intervention participants’ cooking behaviours according to end-of-study interviews are described here. Participants commonly prepared their own breakfast and lunch (usually involving very little hands-on time), because this was “routine” or “our responsibility”. Some were allowed to get their own dinner if they did not like what was on offer, or organise their dinner when parents were away. The most common foods they prepared for themselves only were toast, cereal, sandwiches, noodles, eggs and pasta. The few who prepared something requiring more than minimal input just for themselves did so when they were hungry and food was not being served (“I’m hungry, I make food”), when the normal routine was disrupted because of after-school activities, or during weekends which may be less structured (“it’s a kinda free-for-all kind of weekend”). 

Most participants reported cooking family meals regularly, usually once or twice a week. Pasta was the most common dish prepared for the family (e.g., spaghetti bolognaise), but other meals like satay chicken, treat meals (e.g., pancakes) and baking were often mentioned. Two participants described their cooking as “meat, carbohydrates/rice and vegetables”. When asked whether they helped their parents cook, those answering “no” explained “it’s generally like, you’re cooking, that’s it”. Those replying “yes” mostly helped with preparation, such as peeling and cutting vegetables, but some also made basic meal components like mashed potatoes. Some participants offered to cook family meals because they liked to or wanted to be helpful, however participants usually cooked when asked or told to. This generally occurred ad hoc and reflected fitting into families’ needs on a given night, such when parents were busy, stressed or away. Only one participant had a set cooking day each week, which was based on when parents were out.

Family factors substantially influenced whether participants regularly cooked family meals. Those not cooking regularly felt no need, as parents did this, whereas parents often being away or busy created an opportunity to step up. Those from single-parent households described significant responsibility, comparable to their parent or as the primary food preparer. When parents were away, those with younger siblings often described being asked to cook family meals, while few participants who were only children or had older siblings mentioned being asked this. Reasons some participants did not cook or help were that there was no need (“I don’t really need to cus’ Mum just normally cooks all the time”), and that the meal was already cooked when they got home because young siblings needed to be fed early. Almost all participants who did not like cooking and did not regularly cook still did so occasionally, when told to. 

#### 3.3.3. Cooking from an Adolescent’s Perspective

The facilitators, barriers, likes and dislikes of cooking were identified during end-of-study interviews and are summarised in Table 4. Responses have been combined for control and intervention participants. Cleaning up and choosing what to make stood out as adding significantly to participants’ perceived burden of cooking. The most common dislike about cooking was cleaning up, followed by fussy eating among family members (particularly siblings). 

## 4. Discussion

The COOK Study RCT investigated the immediate and longer-term effects of an adolescent cooking intervention on mental well-being, diet quality, BMI and cooking-related outcomes. Post-intervention increases were seen in the intervention group for mental well-being, diet quality, the frequency of helping with dinner, positive cooking attitude and cooking self-efficacy, which showed the largest effect sizes. The intervention group also showed an increase in BMI z-scores from baseline relative to controls. Only differences in cooking self-efficacy scores remained significant at 12 months. Group interview data suggested that whether adolescents cook, how often they cook and what they make is substantially influenced by household factors, including routines and responsibilities, food availability, and acceptability of foods to family members. 

To our knowledge, the only other cooking intervention investigating adolescent mental well-being was a recent single-group feasibility study, reporting increased well-being scores for most participants [26]. The COOK Study found a statistically significant difference favouring the intervention, although the effect size was small (3; 10 is considered clinically significant) [58]. Other factors, like peer relationships, substantially affect adolescent well-being [59], which the intervention potentially influenced but did not directly target nor assess—although group interview feedback showed participants enjoyed making friends, which could have enhanced self-esteem [60]. Overall, these findings support observational research which has suggested that cooking and mental well-being are related [34], and further research is needed to understand pathways.

Previous adolescent cooking interventions have measured markers of diet quality such as nutrient intakes or consumption of food groups [61], although the COOK Study is the first we know of to apply a diet quality index. Our findings concur with the many other interventions which reported improved aspects of diet quality [61]. Taste preference and availability are two of the biggest predictors of adolescents’ fruit and vegetable intakes [62] and may have been influenced by the intervention, as other cooking interventions have found increased taste preferences for fruit and vegetables [20,28]. Meal kits may have improved the availability of healthy foods, either directly or by leaving more financial resources available for purchasing healthier foods, which may contribute to why the DQI difference between groups was not maintained at follow-up. Given that most adolescent interventions that have included a substantial amount of cooking have focussed on the adolescent and done little to address the family and wider food environments, long-term dietary changes may not be realistic. However, incorporating cooking into multi-component interventions that do target these areas may be a particularly effective way of engaging adolescents in healthy eating, as most enjoy the opportunity to cook [11]. 

At post-intervention, we observed an increase in the intervention group’s BMI z-scores (0.08, *p* = 0.006), despite improved diet quality scores also at this time (although the NZA-FFQ is non-quantitative; serving sizes may have changed). This BMI change equates to an additional extra 680 g and 663 g of body mass for females and males, respectively, for adolescents with a median BMI according to WHO 2007 reference tables and sample median height and age [63]. Greater adolescent cooking involvement has been associated with a higher BMI in observational studies [53,64,65], however only two other cooking intervention controlled trials with older children or adolescents have included BMI as an outcome. One found a reduction in BMI amongst a subgroup classified as overweight at baseline [66]. The other mirrored the COOK Study, with BMI z-scores also increasing post-intervention, but not being sustained at follow-up [67]. As has been suggested by authors reporting on cross-sectional studies, adolescents who cook more often may be more likely to have excess energy intakes or lower levels of physical activity [34,53], for example, by spending time cooking that they would otherwise have spent been physically active, or making and eating more treat foods. 

Adolescent interventions which have included cooking behaviours as an outcome have shown mixed results [13,25,26]. The frequency of COOK participants helping with dinner initially increased by approximately one evening per fortnight more among the intervention group, while the frequency of cooking main meals from basic ingredients did not change, despite receiving meal kits. As the baseline frequency of cooking from basic ingredients was 0.5 times per week, kits could have been absorbed into this to some extent, rather than providing an additional task. Additionally, not all kits may have been made—Facebook posts indicated good adherence for most participants, although this was not specifically measured as we doubted responses would be reliable. 

Cooking self-efficacy has been the most consistently improved outcome of cooking interventions [11]. A key element of these programmes is practical experience, which is the best way to grow self-efficacy according to SCT [7]. Of all the outcomes reported here, we found the largest effect sizes for cooking self-efficacy. Interestingly, the intervention group’s significantly better cooking self-efficacy at follow-up was not accompanied by greater cooking frequency, nor diet quality. During group interviews, participants described more constraints than enablers to cooking at home. This may mean that, for many adolescents, additional self-efficacy growth may not drive behavioural changes after a certain level is reached, although behavioural differences could emerge once participants move into living situations where they have greater food responsibility.

Changes in attitudes towards cooking have been inconsistent amongst studies with adolescent participants [13,15,16,18]. We observed an initial increase in cooking attitude from baseline relative to controls (11 on a scale of 100), with no difference between groups at follow-up. Post-intervention group interviews showed that while the intervention was novel and fun overall, cooking has additional challenges in a family context compared to a teaching kitchen, particularly where siblings are concerned. By end-of-study interviews, cooking was more of a chore and participants had little autonomy. 

Applying their new skills at home is likely important for adolescents and their families to reap the full benefits of cooking interventions. We combined techniques used by previous cooking interventions to try and improve buy-in from families and facilitate cooking at home, by inviting a family member for the last day of COOK week to showcase their adolescent’s capabilities [14,15,16,17,20], and providing meaningful tasks, through the meal kits, that made a contribution to the family [24,39]. Nonetheless, interview data demonstrate that family routines, likes and dislikes strongly influenced what and when participants could cook. Family-based cooking interventions are common with children but less so with adolescents; working with families and home-based components is an aspect which adolescent interventions should develop further.

Cooking intervention research is an emerging area and few adolescent studies have included both a comparison group and a follow-up [27,67]. The COOK study contributed an RCT with a 12-month follow-up, important for differentiating intervention effects from normal development in a young population, and for investigating whether any benefits could be maintained. Another strength was the inclusion of a support phase onto a teaching kitchen-based programme to facilitate participants in building cooking habits, which has not been done before to our knowledge. Including group interviews provided insight into how well the support phase worked and the home dynamics surrounding adolescent cooking. 

These results may not be generalisable, as adolescents who were more interested in cooking may have been more likely to enrol in the study, although baseline cooking ability and involvement statistics were similar to those reported by the nationally representative Youth’12 survey of high school students [34]. Furthermore, if the larger proportion of female to male participants in this study has influenced results, they may be less generalisable to males. This study’s biggest limitation was the small control group sample size, which was a consequence of not meeting recruitment targets and preferentially randomising to the intervention group to maintain a suitable class size, compounded by a high drop-out rate (51%) among the control group, many of whom enrolled in the study specifically to participate in the cooking classes. In cooking intervention research it has been more common to allocate condition by school or class rather than by individual [27,28,29,32]; cluster RCT’s by school or class may improve control group retention, as all students within a peer group receive the same condition. Wait-list controls have been utilised for other cooking interventions [27,28,68], however the follow-up period would probably need to be considerably shorter than 12 months to remain attractive to control participants. Secondly, as there was only one intervention group, the impact of the support phase itself cannot be determined—this aspect was piloted during the COOK Study and was shown to be feasible and acceptable to participants.

Future studies should prioritise cluster RCTs with follow-ups, using larger sample sizes. BMI changes should be further investigated, accounting for the foods adolescents actually cook at home, energy intakes and physical activity. Greater focus on understanding and working with families to facilitate adolescents to cook healthy food may improve the outcomes of cooking interventions, as well as contributing an evidence base from which recommendations for families can be developed. 

## 5. Conclusions

The COOK intervention appeared to have a beneficial effect on most outcomes at first, although BMI also initially increased. Only changes in cooking self-efficacy were maintained at follow-up. Group interviews suggested that whether participants cook more often after a cooking intervention and what they make is heavily influenced by family factors.

## Figures and Tables

**Table 1:** Baseline characteristics of the COOK (Create Our Own Kai) Study participants (*n* = 118).

	Control Group (*n* = 27)	Intervention Group (*n* = 91)
Age (years), mean (SD)	14.3 (0.7)	14.1 (0.8)
Sex (female), *n* (%)	21 (78)	55 (60)
Ethnicity, *n* (%)		
Māori	4 (15)	13 (14)
Pacific	0 (0)	0 (0)
New Zealand European and Other	23 (85)	78 (86)
Socio-economic status ^a^, *n* (%)		
Low (NZDep 8–10)	4 (15)	12 (13)
Medium (NZDep 4–7)	8 (30)	39 (43)
High (NZDep 1–3)	15 (56)	40 (47)
Weight status ^c,d^, *n* (%)		
Healthy weight	16 (59)	55 (63)
Overweight	7 (26)	23 (26)
Obese	4 (15)	10 (11)
BMI z-score ^b^, mean (SD)	0.7 (1.2)	0.4 (1.2)

^a^ Deciles of the New Zealand deprivation index (NZDep [49]) determined by home address. ^b^ New Zealand European and Other. ^c^ Three participants missing BMI z-score, all from the intervention group. ^d^ Defined using the WHO 2007 Growth Reference [57].

**Table 2:** Effect of the COOK intervention on BMI z-score, diet quality, and mental well-being at 7 weeks and 12 months (*n* = 118).

	Control Group		Intervention Group		Mean Difference	*p*-Value
	*n*	Mean (SD)	*n*	Mean (SD)	(95% CI) ^a^	
Mental well-being ^b^						
Baseline	27	65 (14)	90	69 (19)		
Change at 7 weeks	26	−1 (14)	84	1 (19)	3 (1, 5)	0.005
Change at 12 months	27	−2 (17)	85	−4 (21)	−1 (−10, 7)	0.762
Diet quality ^c^						
Baseline	26	57 (14)	88	60 (14)		
Change at 7 weeks	26	−2 (12)	82	2 (13)	4 (0.2, 8)	0.041
Change at 12 months	26	−2 (9)	81	−2 (13)	1 (−5, 7)	0.751
BMI z-score ^d^						
Change at 7 weeks	26	0.00 (0.18)	85	0.07 (0.17)	0.08 (0.02, 0.14)	0.006
Change at 12 months	27	0.05 (0.44)	85	0.07 (0.40)	0.02 (−0.002, 0.04)	0.076

^a^ From a mixed effects regression analysis with random effects for participant and intervention stream. ^b^ Using the WHO-5 Well-being Index [50]. Scale range is from 0 to100. ^c^ Using the NZA-DQI Diet Quality Index [52]. Scale range is from 0 to 100. ^d^ Defined using the WHO 2007 Growth Reference [57].

**Table 3:** Effect of the COOK intervention on cooking skills, attitudes, and self-efficacy at 7 weeks and 12 months (*n* = 118).

	Control Group		Intervention Group		Mean Difference	*p*-Value
	*n*	Mean (SD)	*n*	Mean (SD)	(95% CI) ^a^	
Helps with dinner, times/week						
Baseline, median (25th, 75th percentile)	26	1 (0, 2)	88	1 (0.5, 2)		
Change at 7 weeks	25	0.4 (1.5)	82	0.7 (1.5)	0.4 (0.1, 0.6)	0.001
Change at 12 months	26	0.1 (1.0)	82	0.2 (1.3)	0.1 (−0.1, 0.3)	0.455
Cooks main meal, times/week						
Baseline, median (25th, 75th percentile)	27	0.5 (0.5, 1)	91	0.5 (0, 1)		
Change at 7 weeks	26	0.5 (0.8)	84	0.7 (1.7)	0.2 (−0.6, 0.9)	0.646
Change at 12 months	27	0.3 (1.0)	86	0.2 (1.7)	0.0 (−0.4, 0.4)	0.931
Self-efficacy for specific cooking tasks & techniques ^b^						
Baseline	27	63 (21)	91	53 (17)		
Change at 7 weeks	26	−1 (12)	85	26 (15)	28 (17, 38)	<0.001
Change at 12 months	27	2 (16)	86	22 (17)	19 (12, 26)	<0.001
General cooking self-efficacy ^b^						
Baseline	27	66 (16)	91	64 (17)		
Change at 7 weeks	26	2 (10)	85	16 (13)	15 (12, 18)	<0.001
Change at 12 months	27	4 (13)	86	12 (16)	9 (5, 7)	<0.001
Positive cooking attitude ^b^						
Baseline	27	67 (19)	91	69 (17)		
Change at 7 weeks	26	−3 (10)	85	8 (14)	11 (7, 15)	0.001
Change at 12 months	27	−2 (17)	86	−1 (17)	2 (−1, 5)	0.278

^a^ From a mixed effects regression analysis with random effects for participant and intervention stream. ^b^ Scale range is from 0 to 100.

**Table 4:** Factors influencing participants’ experiences of cooking at home during end-of-study interviews.

Factor	Summary	Comments
Feeling like it	Participants mostly cooked when they were asked to, although they did volunteer to cook sometimes as well. Often participants were asked to cook when they did not feel like it.	A reason some did feel like cooking was that this meant they could choose what they felt like eating for the meal. However, this factor was seldom mentioned, possibly because most participants could not choose the meal (see below). Reasons participants may not feel like cooking included being tired, having other things they wanted or needed to do, or disliking being interrupted from something they were already doing.
Choosing what to cook	Although the decision for what to cook for the family might be influenced by what participants felt like, it was ultimately bound by: - ingredient availability, and - acceptability to family members. This usually meant cooking from a family repertoire. Most participants felt they had more choice when cooking for themselves, as they need not accommodate others’ preferences.	Meals were often based on leftovers or fresh foods that needed using (“anything in the fridge”), although some participants had input into grocery purchase decisions (“if I’ve got an idea that I wanna cook and stuff, I’ll give them what I want them to buy and if they’ve got the money they’ll get it”). Virtually all participants said family dishes were constrained by what others would eat, particularly siblings. Parents ensured suitable choices by telling participants what to make or vetting their choice. Most participants agreed “getting told is a lot easier” than choosing what to cook for the family, as finding something they both had ingredients for and everyone would eat was difficult, and frustrating if they could not make what they wanted. Only a few participants preferred to choose what to cook, because “then I know I’ll get to eat it when I when I’m done and it’s nice”. Those who had tried commercial meal kit services appreciated the straightforwardness of having the choice made for them and ingredients and recipes provided. Although most participants felt they had more choice when cooking for themselves only, it was unclear how much freedom was actually exercised, given their convenience requirements and the limited range of foods they described making most often (e.g., noodles, toast).
Effort required	Participants generally wanted to cook something quick and easy. Cooking for the family often involved greater effort than cooking for themselves.	Some felt they put “about the same amount” of effort into cooking for themselves versus for others. One participant even used cooking for them self as an opportunity to experiment. Other participants had a stronger convenience orientation when cooking for themselves (“when I’m cooking for myself it’s generally a lot quicker because I got other things to do and, like, I’m hungry, I need to get something quick, done and then get back to doing whatever I want”). Cooking a family meal often meant more time and effort, particularly with presentation (“well if it looks good they’re gonna eat it, it makes sense!”), and for some constituted an “actual meal”.
Cooking the food	Some participants described enjoying alone time, autonomy and creativity while cooking. Less enjoyable aspects were the time it takes. Cooking was easier when there were clear instructions and family members cooperated.	Enjoyable aspects of cooking were having some alone time and exercising control over their environment, which included playing their choice of music or having some quiet. In some cases, being the cook endowed authority to tell others to go away (“[I’m] independent and bossy!”). One participant described a sense of achievement when cooking, while others enjoyed getting creative, especially with presentation. Dislikes included long repetitive tasks like chopping, which could be boring, using an unreliable oven, and being stuck looking after food (“yeah waiting for things to cook and cus’ you just want to go off and do something else but if you do that you might forget and you’ll burn, boil over or whatever”). Some participants had help from family, particularly when cooking for lots of people or when things were not going well, such as peeling and cutting, verbal direction and sometimes stirring. Cooking was easier with clear instructions on how long to cook something, rather than having to guess, and if family members were helpful or well-behaved if they came into the kitchen.
Family responses	Positive family responses showed support, appreciation, enjoyment, and helping. Family appreciation could also make participants feel self-conscious. Family members (particularly siblings) who disliked or refused to eat food was a barrier.	Positive family reactions included parents being appreciative and supportive, and siblings enjoying the food. Other positive behaviours were family members being cooperative during cooking, either by staying out of the way or assisting, and helping clean up. Some participants described feeling awkward when family members made a big deal of them cooking (Participant: “Um I think if its someone other than mum cooking they feel pressure to be extra nice about it.” Facilitator: “Does that perception that they are trying to be extra nice make you feel better or worse?” Participant: “No, I find it off-putting”). The most common complaint relating to families was siblings not liking or refusing to eat what participants had cooked.
Cleaning up	Cleaning up was the most common dislike about cooking. Cooking family meals did not usually mean being “off” dishes—in many cases it guaranteed another chore.	Some participants had to clean anything they dirtied (“everything in my house is kinda if you make a mess then you have to clean it up”). For others, whether they cooked was unrelated to whether they did dishes, as they always did dishes anyway or took turns with siblings. Arrangements where the person who cooks does not do dishes were a minority, and included participants doing dishes as they go but someone else doing the big pots at the end.
